# Enhanced Toluene Combustion over Cryptomelane Catalysts: Influence of Cu Doping on Physicochemical Properties and Catalytic Performance

**DOI:** 10.3390/ma19010159

**Published:** 2026-01-02

**Authors:** Jakub Mokrzycki, Joanna Kryściak-Czerwenka, Dorota Duraczyńska, Mateusz Marzec, Robert Karcz

**Affiliations:** 1 AGH University of Krakow, Faculty of Energy and Fuels, al. A. Mickiewicza 30, 30-059 Krakow, Poland; 2 Jerzy Haber Institute of Catalysis and Surface Chemistry, Polish Academy of Sciences, Niezapominajek 8, 30-239 Krakow, Poland; joanna.krysciak-czerwenka@ikifp.edu.pl (J.K.-C.); dorota.duraczynska@ikifp.edu.pl (D.D.); robert.karcz@ikifp.edu.pl (R.K.); 3 AGH University of Krakow, Academic Centre for Materials and Nanotechnology, al. A. Mickiewicza 30, 30-059 Krakow, Poland; marzecm@agh.edu.pl

**Keywords:** cryptomelane, toluene combustion, oxidation, copper-doped catalysts

## Abstract

The catalytic combustion of toluene over cryptomelane and Cu-doped cryptomelane catalysts was investigated to evaluate the effect of copper incorporation on catalytic performance. It was found that a small addition of Cu into the cryptomelane framework resulted in a notable decrease in the temperature of 90% toluene conversion. To elucidate the structure–activity relationship, the catalysts were comprehensively characterized using XRD, FTIR, SEM-EDS, N_2_ physisorption, XRF, XPS, O_2_-TPD, and H_2_-TPR techniques. The results revealed that Cu doping modifies the physicochemical properties of cryptomelane, most importantly the share of lattice and surface oxygen species, enhancing redox behavior and oxygen mobility, which in turn improves catalytic activity. For the lowest dose of Cu, both the temperatures of 50 and 90% conversion were found to be lowest among investigated catalyst series: 180 and 195 °C, respectively. These findings highlight the potential of Cu-doped cryptomelane as an efficient catalyst for the abatement of volatile organic compounds.

## 1. Introduction

One of the contemporary challenges of environmental protection is the mitigation of constantly growing emissions of volatile organic compounds (VOCs) into the atmosphere, which are considered impactful air pollutants worldwide [1]. Due to their high vapor pressure, relatively low boiling points (50–260 °C), and low solubility in water, their transfer to the atmosphere is significant, causing inter alia photochemical fog, ozone depletion, and acid rain [2]. Among VOCs, toluene is considered one of the most meaningful air pollutants, owing to its toxicity, relatively high chemical stability, and broad industrial application. Its main sources involve anthropogenic activity, which involves the industrial production of glues, organic dyes, organic solvents, and pharmaceuticals.

Among various toluene neutralization methods, its catalytic combustion has gained attention, owing to the opportunity to convert toluene into CO_2_ and water vapor, which are considered as non-toxic compounds. What is more, the process can be conducted at relatively moderate temperatures (200–300 °C). The key role within this process is governed by the catalysts, whose physicochemical properties provide activity, selectivity, and stability. The general mechanism of toluene combustion according to the Langmuir–Hinshelwood model assumes adsorption of toluene and oxygen over the catalysts’ surface, in which the rate controlling step is the surface reaction. Next, the desorption of combustion products occurs [3]. However, the most widely suggested mechanism of the VOCs combustion is the Mars-van Krevelen model, in which it is assumed that the catalysts lattice oxygen species takes place in the reaction forming vacancies, which are then filled by gaseous oxygen. Simultaneous occurrence of both mechanisms is also considered, where Langmuir–Hinshelwood occurs at lower process temperatures, whereas Mars-van Krevelen occurs at higher process temperatures [4]. In recent years catalysts based on mixed manganese oxides like cryptomelane (OMS-2) have gained attention owing to their unique surface properties and a high share of lattice oxygen. Such presence of lattice oxygen favors formation of oxygen vacancies and increases the mobility of lattice oxygen, which promotes the reaction at relatively low temperatures (about 200–250 °C) [5].

OMS-2 is characterized by a unique tunnelable crystalline, needle-like structure forming a double 2 × 2 octahedral molecular sieve from MnO_6_ building units. The stoichiometric formula of OMS-2 is K_x_Mn_8_O_16_⸱nH_2_O [6]. Its channels are of size 0.46 nm, in which exchangeable cations can occur: H_3_O^+^, Na^+^, K^+^, Mg^2+^, Ca^2+^. The presence of alkali cations stabilizes the structure and compensates for the negative charge within the material. The key feature of cryptomelane is coexistence of manganese of various oxidation states (Mn^2+^, Mn^3+^, and Mn^4+^). The ability to change and tune the oxidation state of manganese ions via changing stoichiometry ratio of K/Mn/O and doping with d-block or f-block metals at the synthesis step made cryptomelane an interesting material for catalytic applications. What is more, the synthesis of cryptomelane is considered as simple and it utilizes relatively environmentally friendly ingredients and can be conducted under mild temperature conditions (about 100 °C). The most common methods of OMS-2 production are reflux synthesis [7] and hydrothermal synthesis [8]; however, sol–gel methods were also reported [9,10].

Cryptomelane catalysts have been already reported in the literature as toluene combustion catalysts [11]. It is known that the mixed valence state of manganese-based catalysts improves redox properties and allows formation of oxygen vacancy defects, which are highly active in combustion of VOCs [12]. In the work of Zhang et al. [13], cryptomelane with Mn^3+^/Mn^4+^ ratio of 1.201, and which shares O_ads_/O_latt_ oxygen species (adsorbed to lattice oxygen ratio) of 0.384, which were the highest among the investigated series of toluene combustion catalysts, have demonstrated the lowest temperature of 50% conversion at 225 °C and 90% conversion at 238 °C. Hence a high share of adsorbed, loosely bounded oxygen species can play an important role in the oxidation of toluene. The redox properties of cryptomelane are known to be tuned by simple introduction of dopants to its framework (Ag [14], Ce [15], Co [16], Cu [17], Fe [18], V [19]). Among the abovementioned, Cu has been reported to effectively weaken the Mn-O bonds in Mn-O-Cu bridges causing an increase in the active oxygen species mobility, or by replacing Mn^2+^ with CuO_6_ octahedra, causing distortion in the crystal lattice, which results in the generation of crystal structural defects [20].

The aim of the present study was to investigate the catalytic activity of cryptomelane catalysts doped with copper in order to clarify the effect of copper substitution and structural changes in the catalyst on the catalytic activity in the combustion of toluene reaction. Prepared materials were characterized in detail to gain a better understanding of the physicochemical properties that can influence the catalytic properties using the following methods: XRD, FTIR, SEM-EDS, N_2_ physisorption, XPS, XRF, diffuse reflectance UV-vis (DR/UV-vis), and H_2_-TPR, O_2_-TPD. The main outcome of this research was the demonstration of the potential of cryptomelane catalysts doped with low copper loading as sustainable and efficient materials for mitigation of VOCs in air, using toluene as a model compound.

## 2. Materials and Methods

### 2.1. Synthesis of Cryptomelane and Cu-Doped Cryptomelane

Cryptomelane (OMS-2) was synthesized according to the following procedure. Briefly, 5.07 g of MnSO_4_·H_2_O (Sigma-Aldrich, St. Louis, MO, USA) was dissolved in solution containing 1.8 mL of HNO_3_ (Avantor, Gliwice, Poland) in 18 mL of distilled water (Solution A). In a separate beaker, 3.51 g of KMnO_4_ (Sigma-Aldrich, St. Louis, MO, USA) was dissolved in 60 mL of distilled water (Solution B). Solution A was poured into 250 round bottom flasks, while Solution B was added dropwise under constant stirring and heating at ~100 °C under reflux. After 24 h, the obtained solid product was filtrated, washed with deionized water and dried overnight at room temperature. Next, it was placed in over for 12 h at 120 °C, followed by calcination at 500 °C for 4 h. The obtained dark-brown powder was denoted OMS-2. The Cu-doped materials were prepared by adding to Solution A an appropriate amount of Cu(NO_3_)_2_ (Avantor, Gliwice, Poland): 0.104, 0.198, or 0.992 g. Further procedure was completed as described above. The obtained Cu-doped materials were denoted as CuX-OMS-2, where X = 1,2, or 3, and represents the increasing dose of copper salt used during the synthesis.

### 2.2. Materials Characterization

The crystalline phase composition was determined by means of PANalytical Empyrean diffractometer (Malvern Panalytical, Malvern, UK). The source of radiation was CuKα λ = 1.5406 Å, and the measurements 2θ range varied from 10 to 80°.

Transmission Fourier transform infrared (FTIR) spectra were recorded using a Nicolet 6700 (Thermo Scientific, Madison, WI, USA) spectrometer under atmospheric conditions, in the 4000–400 cm^−1^ range, at a spectral resolution of 2 cm^−1^ for samples prepared as KBr disks. The sample–KBr ratio for disks preparation was 1:100.

SEM-EDS was employed to show the surface morphology of the obtained catalysts. Prior to the imaging, samples were coated with a 30 nm layer of chromium using K575X Turbo Sputter Coater (Quorum Emitech, South Stour Avenue, Ashford, UK). Images were collected using JEOL JSM 7500 F (JEOL, Tokyo, Japan) equipped with an energy-dispersive X-ray spectroscopy system, AZtecLiveLite Xplore 30 (Oxford Instruments, Abingdon, UK).

The diffuse reflectance spectroscopy (DR/UV-vis) was performed using (Evolution One/One Plus UV-vis Spectrophotometer, Thermo Fisher Scientific Inc., London, UK) at wavelengths from 190 to 700 nm with a scan rate of 200 nm min^−1^. The results were demonstrated as Kubelka–Munk function.

The low-temperature N_2_ adsorption at −196 °C was performed to determine the specific surface area (S_BET_) of investigated catalysts using ASAP 2020 physisorption analyzer (Micrometrics, Norcross, Georgia, USA) and calculated from the Brunaer–Emmett–Teller method.

The bulk chemical composition of the OMS-2 materials was determined using X-ray fluorescence spectroscopy (XRF). ARL QUANT’X spectrometer (Thermo Scientific, Waltham, MA, USA) with a rhodium anode (4–50 kV in 1 kV increments) and beryllium windows was employed. A 1 mm beam and a 3.5 mm Si(Li) drifted crystal detector with Peltier cooling (at about −88 °C) were used for all measurements. Quantitative analysis of elements within the catalysts was performed using UniQuant software (Version 3, Thermo Fisher, West Palm Beach, FL, USA) and metallic calibration standards.

The XPS analyses were carried out in a PHI VersaProbeII Scanning XPS system using monochromatic Al Kα (1486.6 eV) X-rays focused to a 100 µm spot and scanned over the area of 400 µm × 400 µm. The photoelectron take-off angle was 45° and the pass energy in the analyzer was set to 117.50 eV for survey scans and 46.95 eV to obtain high-energy resolution spectra for the C 1s, O 1s, Cu 2p, Mn 2p, and K 2p regions. A dual beam charge compensation with 7 eV Ar^+^ ions and 1 eV electrons was used to maintain a constant sample surface potential regardless of the sample conductivity. All XPS spectra were charge referenced to the unfunctionalized, saturated carbon (C-C) C 1s peak at 285.0 eV. The operating pressure in the analytical chamber was less than 2 × 10^−9^ mbar. Deconvolution of spectra was carried out using PHI MultiPak software (v.9.9.3). Spectrum background was subtracted using the Shirley method.

Oxygen temperature programmed desorption (O_2_-TPD) was performed to gain a better understanding of the oxygen mobile forms within the OMS-2 catalysts. Experiments were performed in a quartz U-shape tube reactor (Micromeritics, Norcross, GA, USA). Apparatus is equipped with a moisture trap. Prior to the measurement, about 50 mg of sample was calcined at 500 °C for 1 h in air flow of 50 mL min^−1^. Next, sample was heated from room temperature to 800 °C with a heat ramp of 5 °C min^−1^ in He flow of 50 mL min^−1^. Gases were detected by TCD.

The reductivity of the samples was determined by means of H_2_-TPR method using a programmed warming chemisorption analyzer (Micromeritics, Norcross, GA, USA). About 30 mg of sample was placed in U-tubes and heated from room temperature to 500 °C temperature at 10 °C min^−1^ heating ramp and reduced in a 10% H_2_/Ar gas stream. Gases were detected by TCD.

### 2.3. Catalytic Oxidation of Toluene

Toluene catalytic oxidation was performed in a fixed-bed quartz reactor. Prior to the test, the catalysts were pressed into disks, ground, and sieved to obtain a fraction 0.3–0.5 mm. The temperature range of the catalytic tests was from 150 to 250 °C. Before the reaction, the catalyst was activated for 1 h at 350 °C in the pure air flow. After cooling down, toluene (500 ppm in air) was fed into the reactor at 20,000 GHSV ml⸱g^−1^⸱h^−1^ (total flow of 84 mL min^−1^). After reaching each reaction temperature, the reactor was stabilized for 30 min. Samples were collected using 1000 μL Hamilton gastight syringe at the reactor inlet and outlet. Gas composition was analyzed using Perkin-Elmer Clarus 500 GC system (Perkin-Elmer, Shelton, CT, USA) equipped with Elite-1 column (30 m, 0.32 mm ID, 3 μm df), FID detector, and methanizer. Calibration of GC signals was performed using toluene/CO_2_/air mixtures of known composition. For each temperature at least three separate analyses for substrate and products were repeated. Stability tests were performed by leaving the catalyst at the desired temperature (close to 50% and 90% conversion) and monitoring the changes in activity of the catalyst.

Conversion of toluene was calculated using Equation (1):

(1) $$X_{tolene}=\frac{C_{tol,s}-C_{tol,p}}{C_{tol,s}}$$

where C_tol,s_ and C_tol,p_ are measured concentrations of toluene in the stream at the reactor inlet and outlet. For each temperature, the carbon balance for the gas stream was calculated, showing that CO_2_ is the main product of the toluene combustion.

## 3. Results and Discussion

**Figure 1:**
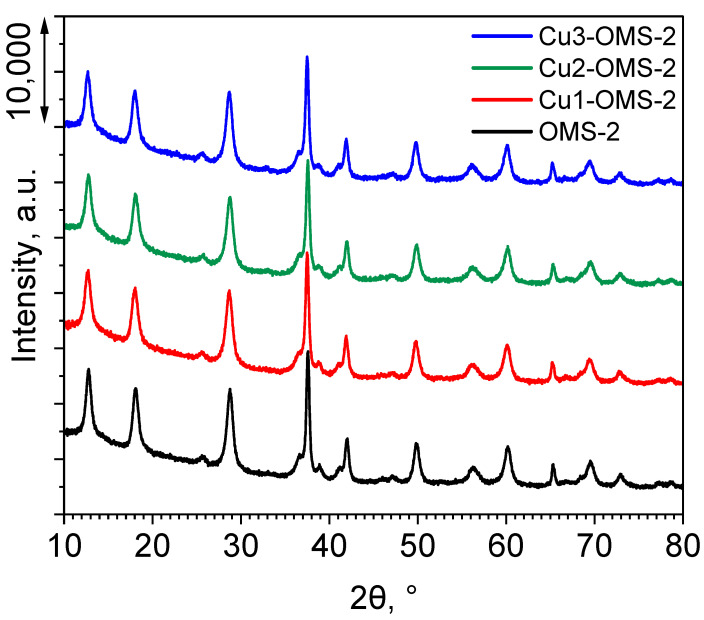
XRD profiles of investigated catalysts.

The XRD profiles of the investigated catalyst series are presented in Figure 1. For all the materials, characteristic reflexes appeared, which were consistent with the (PDF 44-1386), implying a successful synthesis of OMS-2 material [17,21,22]. The reflection peaks at specific 2θ values can be identified and the corresponding *hkl* Miller’s indices were attributed: 12.698° (1 1 0), 18.000° (2 0 0), 25.672° (2 2 0), 28.727° (3 1 0), 36.514° (4 0 0), 37.489° (2 1 1), 38.867° (3 3 0), 41.090° (4 2 0), 41.883° (3 0 1), 49.748° (4 1 1), 56.157° (6 0 0), 60.096° (5 2 1), 65.309° (0 0 2), 69.482° (5 4 1), and 72.849° (3 2 1). Introduction of copper during the synthesis stage did not influence the XRD pattern of the Cu-doped materials in comparison to pristine OMS-2 material. Also, no additional reflections originating from Cu species were observed, which can be due to the low amount of copper within the CuX-OMS-2 (X = 1,2,3) being below the detection limit of the apparatus or fine dispersion of Cu within the material.

**Figure 2:**
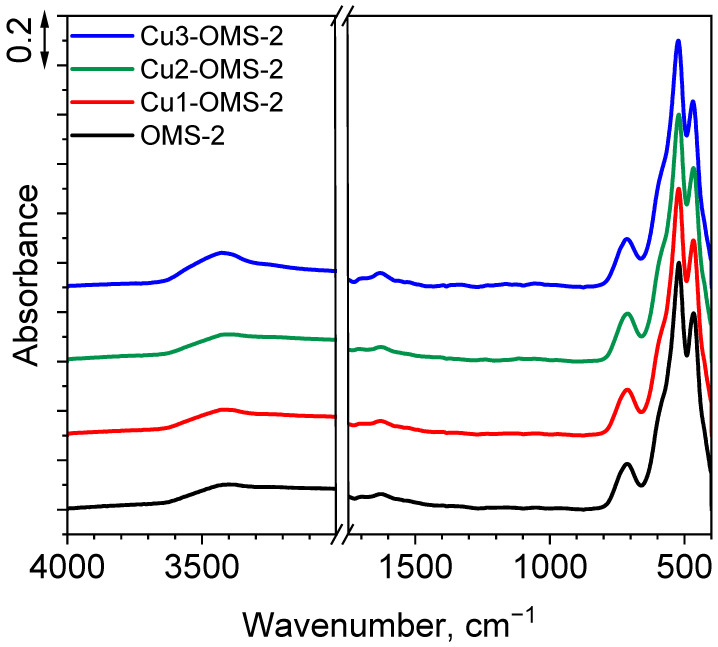
FTIR spectra of investigated catalysts.

To gain a better understanding of the functional groups within the materials, FTIR measurements were conducted. The obtained spectra are presented in Figure 2. For all the materials, five peaks were identified at 3417, 1624, 702, 523, and 467 cm^−1^. First peak can be assigned as O-H stretching vibrations from water molecules adsorbed to the material. Its intensity slightly increased along with increasing dose of copper introduced to the material, which is related to increased hydrophilicity of the CuX-OMS-2 materials (X = 1,2,3). Also, the band at 1624 cm^−1^ is related to presence of bending O-H vibrations from water molecules over the material’s surface. Three wide bands at 702, 523, and 467 cm^−1^ are assigned to the MnO_6_ octahedral framework of cryptomelane (resulting from bending Mn-OH vibrations) as reported by Zong et al. [23]. No additional vibrations were observed for CuX-OMS-2 materials (X = 1,2,3), which can be related to low amount of copper within the obtained series and does not allow to track the increase in the share of oxygen vacancies over the catalysts’ surface [24].

**Figure 3:**
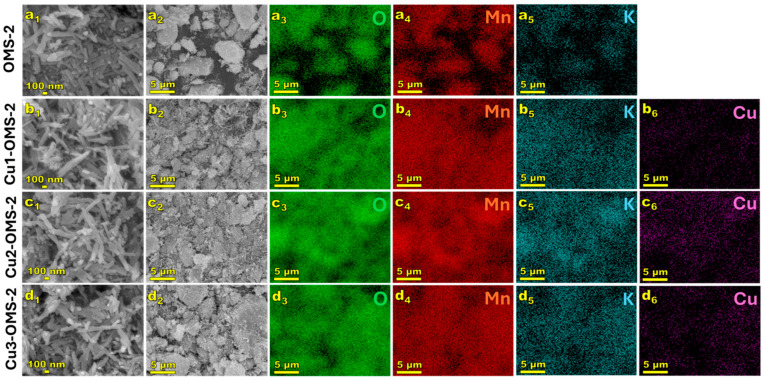
SEM images of investigated samples: OMS-2 (**a**), Cu1-OMS-2 (**b**), Cu2-OMS-2 (**c**), and Cu3-OMS-2 (**d**), where 1—sample magnification of 50,000×, 2—sample magnification of 2500×, 3—oxygen EDS map, 4—manganese EDS map, 5—potassium EDS map, 6—copper EDS map.

SEM images were taken to demonstrate the morphology of the obtained materials and to examine the distribution of elements on the catalysts’ surface. The results are presented in Figure 3. As observed in the images at 50,000× magnification, all the investigated OMS-2 materials exhibited needle-like structures, consistent with the previously published reports [25,26]. The needles were approximately 53 nm wide and >200 nm in length. Introduction of copper into the CuX-OMS-2 (X = 1,2,3) materials can be confirmed, with rather uniform distribution of Cu over the catalysts’ surface. Introduction of copper did not affect the surface morphology of the CuX-OMS-2 catalyst series and can be related to replacement of Mn by Cu within the material’s lattice.

Chemical composition of the materials was investigated by means of XPS and XRF analyses. Summarized XPS results are presented in Figure S1 and Table S1, and the normalized chemical composition is presented in Table 1. From the obtained XRF results, the chemical formula of the catalysts can be defined as K_x_Mn_8-y_Cu_y_O_18_, where x = 0.79–0.88 and y = 0.06–0.21. What is noteworthy is that the results obtained from XRF analysis demonstrated strong correlation with the ones obtained from XPS, implying successful introduction of copper into the OMS-2 both in the bulk and on the surface. Slightly greater values of Cu/Mn ratios as obtained from XPS analysis of Cu-doped OMS-2 materials can suggest occurrence of copper species over the external surface of the material (Table 1). The specific surface area of the materials varied in the range from 58.6 to 66.9 m^2^ g^−1^, for which it was difficult to determine a significant trend guiding the changes in the S_BET_ values. In general, the S_BET_ values were lower than that obtained for parent OMS-2 from 3.0 to 12.4%.

**Figure 4:**
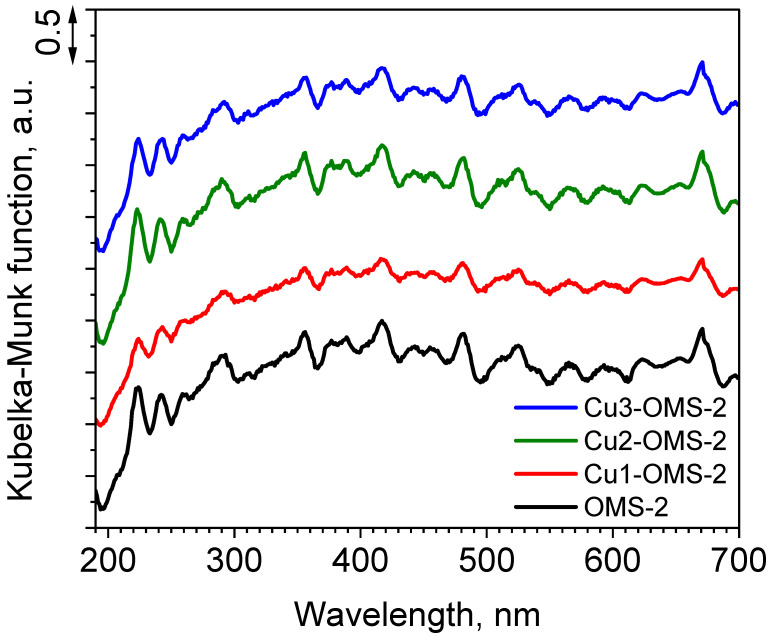
DR/UV-vis spectra of investigated catalysts.

The measurements of UV-vis light absorption by the cryptomelane materials are demonstrated in Figure 4. It can be seen that despite the introduction of copper, the light absorbance in the measured spectrum from 190 to 700 nm demonstrated comparable profile to parent OMS-2. Such phenomenon can suggest that copper introduced into the materials did not cause significant changes to the material’s morphology (as was also confirmed by SEM imaging—see Figure 3) and to its light absorption properties. According to the DR UV-vis spectrum of cryptomelane reported by Sarmah et al. [27], few maxima located in the range from 220 to 350 nm (different types of Mn^n+^ species), and from 350 to 725 (weak d-d band absorption), can be identified. In the obtained spectrum such maxima can be identified at 224, 292, and 670 nm; however, there are numerous light absorption responses which were obtained for all investigated materials. Such light absorption in both UV and visible light regions was also reported to be due to charge transfer in Mn^2+^, Mn^3+^, Mn^4+^, and O^2−^ in an octahedral field [28].

**Figure 5:**
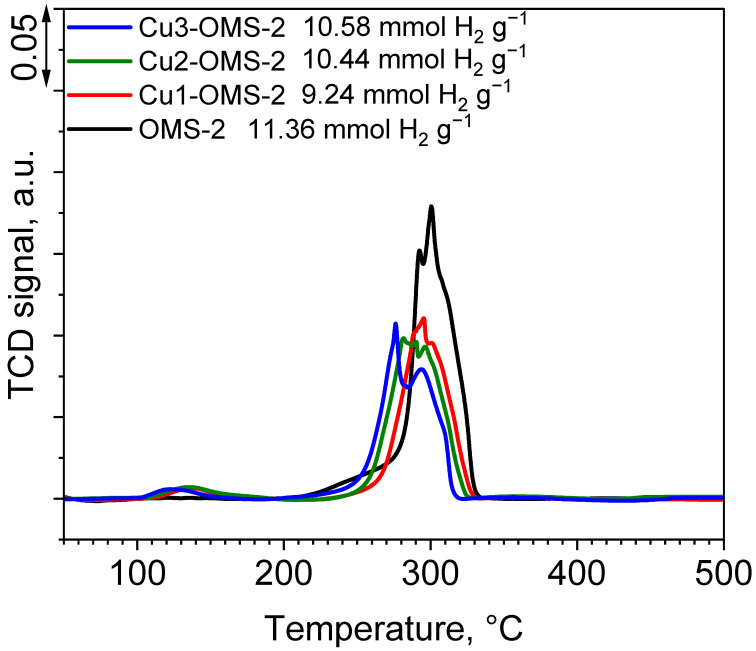
H_2_-TPR profiles of investigated catalysts and H_2_ consumption values.

The profiles of H_2_-TPR tests are presented in Figure 5 and are consistent with the already published references [26,29]. The pristine OMS-2 exhibited three main reduction stages at about 250, 292, and 300 °C and is classified through step by step reduction of manganese species MnO_2_→Mn_2_O_3_→Mn_3_O_4_→MnO, respectively. Introduction of copper ions during the synthesis step caused a shift in the maximum reduction temperature towards lower temperatures (276 °C for Cu3-OMS-2) along with a growing content of Cu within the material. Also, the intensity of the reduction peak was lower, implying occurrence of interactions of introduced copper with manganese octahedra. It can also be observed that a reduction peak occurred for all Cu-doped series at about 125 to 138 °C, which was classified as finely dispersed CuO, which proves occurrence of labile and easily reducible Cu^2+^-O-Mn^x+^ species as was demonstrated in the work of Davó-Quiñonero et al. [20]. Occurrence of low temperature peaks can be explained by Pauling’s electronegativity scale, for which Mn^4+^ has value of 2 and Cu^2+^ value of 1.5, causing electron delocalization effect and activation of O in Cu^2+^-O-Mn^x+^ species [29]. The values of H_2_ consumption obtained for the investigated series are consistent with those reported in the literature [30]. Pristine OMS-2 material exhibited H_2_ consumption of 11.36 mmol g^−1^. Introduction of copper into OMS-2 structure caused slight drop in H_2_ consumption, obtaining the lowest value for Cu1-OMS-2 (9.24 mmol g^−1^). Further increase in Cu doping caused a rise in the consumption value up to 10.58 mmol g^−1^ (Cu3-OMS-2). Shifts in the values can be caused by various oxygen mobility within the investigated materials [31].

**Figure 6:**
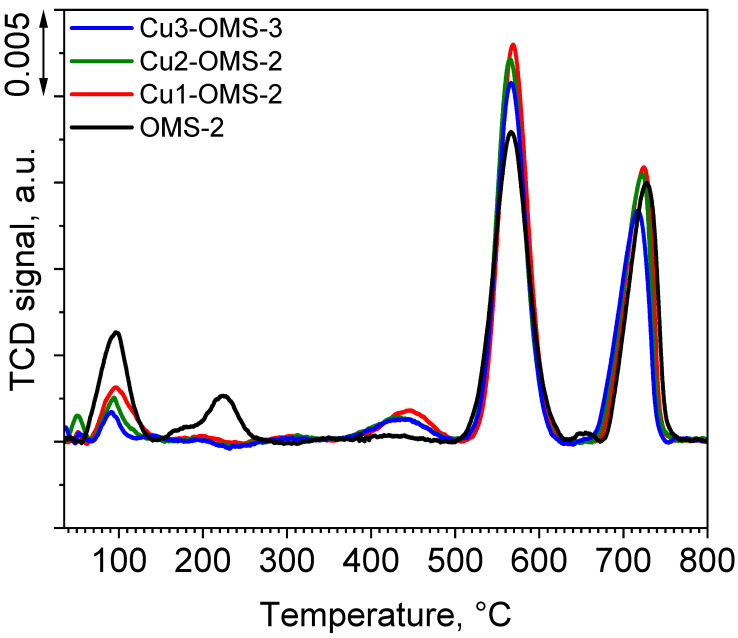
O_2_-TPD profiles of investigated catalysts.

O_2_-TPR profiles of the investigated catalyst series are presented in Figure 6. The pristine OMS-2 catalyst exhibited four desorption peaks with maximum at 98, 226, 568, and 730 °C. First peak can be assigned to both weakly adsorbed molecular oxygen as well as to surface oxygen species (O_surface_). The second one is related to chemically adsorbed oxygen (O_adsorbed_) [13,32,33]. Peaks at 568 and 730 °C were identified as oxygen released from the framework (O_lattice-surface_ and O_surface-bulk_) [13]. It can be seen that introduction of copper during the synthesis stage caused noticeable changes in the share of oxygen species released during the O_2_-TPD process. The share of loosely bonded oxygen species released at lower temperatures lowered with increasing content of Cu within the material. However, the share of the O_lattice-surface_ species was significantly higher when the Cu loading decreased. Also, the highest amount of O_lattice_ species was obtained for Cu1-OMS-2, implying changes in the chemical composition of the obtained catalyst. An additional release of oxygen was observed for all the Cu-doped samples and was located at around 450 °C and was also the greatest for Cu1-OMS-2, hence implying improved surface oxygen spillover of the Cu-doped materials [34].

**Figure 7:**
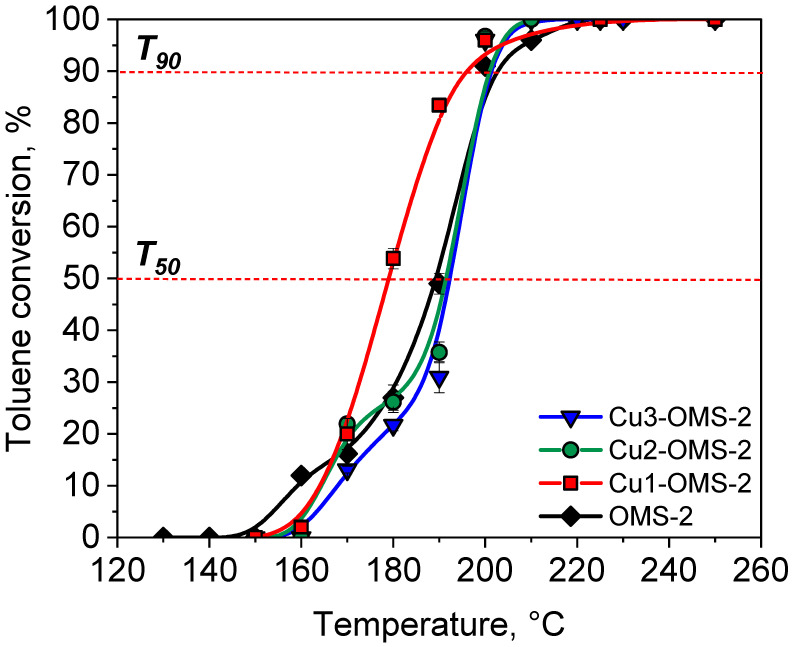
Toluene conversion over investigated catalytic systems.

The results of toluene combustion over investigated catalysts are presented in Figure 7. The carbon balance during toluene combustion over investigated catalysts was summarized in Table S2. All the investigated catalysts start to be active at 160–170 °C. It can be seen that the parent OMS-2 catalyst represented the fastest start of the reaction with a conversion of about 12%. Such phenomenon can be caused by greater share of loosely bonded oxygen molecules for OMS-2 material, which could promote initial activity of the catalyst, as evidenced by O_2_-TPD (Figure 6). At 170 °C all catalysts showed activity from 13 to 20%. The Cu-doped materials (Cu1-OMS-2 and Cu2-OMS-2) were at this temperature more active than OMS-2 and Cu3-OMS-2. Further increase in the reaction temperature showed great differences between the oxidation of toluene, for which Cu1-OMS-2 showed >50% toluene conversion at about 180 °C, whereas for other catalytic systems, this value was obtained >190 °C. Also, the T_90_ (temperature of 90% toluene conversion) was obtained at the lowest temperature for Cu1-OMS-2 catalyst. At 210 °C all investigated catalysts demonstrated 100% toluene conversion. It can be hence concluded that the activity of the catalysts was, rather, not related to the specific surface area (see Table 1), as despite possessing the lowest S_BET_ of 58.6 m^2^ g^−1^, Cu1-OMS-2 was the most active catalyst among the investigated series. The major differences appeared in O_2_-TPD profiles, for which the share of surface oxygen was evidenced to be the highest for Cu1-OMS-2. These oxygen species do not directly take place in this reaction as their desorption temperatures were found to be much above the toluene combustion temperatures (568 and 730 °C). Herein presented combustion temperatures (T_90_) of the investigated samples are just 20 °C higher than those reported in work of Jarczewski et al. [35], where Pt-based ceria oxide catalysts were investigated, implying an opportunity to obtain sufficient catalytic activity for noble metal-free catalytic systems.

The improved activity of Cu1-OMS-2 catalytic system in comparison to parent OMS-2 can be a result of redox properties activation by doping Cu into the cryptomelane lattice and formation of Cu-O-Mn, causing an increase in the share of lattice oxygen species [36,37], which was also evidenced by O_2_-TPD measurement. Santos et al. [38] have found that it is the lattice oxygen species and adsorbed oxygen atoms that govern the oxidation of VOCs. What is noteworthy is the fact that a greater dose of Cu introduced to the synthesis caused a small drop in the activity and catalytic performance at early process stage < 190 °C. Hence, there can be found an optimal Cu dopant dose, which favors activity of the catalyst and stays in line with the results demonstrated by Wantala et al. [39]. According to the XPS measurement (Table S1), the greatest share of O_surface_ and O_adsorbed_ oxygen species was obtained for Cu1-OMS-2 which contributed 24.33% and showed the highest activity in toluene oxidation at temperatures from 160 to 190 °C. Introduction of Cu caused changes in heterogeneity of the surface structure by enhancing O_surface_ oxygen species contributing to enhanced catalytic performance of Cu1-OMS-2 [40,41].

A comparison of performance of various catalytic systems in toluene oxidation is summarized in Table 2.

## 4. Conclusions

The catalytic combustion of toluene over cryptomelane (OMS-2) and Cu-doped OMS-2 catalysts demonstrated that copper incorporation has a significant influence on catalytic performance. All catalysts became active in the temperature range of 160–170 °C, with the parent OMS-2 exhibiting the fastest initial response, likely due to its higher fraction of loosely bound oxygen species. However, Cu1-OMS-2 surpassed the parent material and other Cu-doped variants at elevated temperatures, achieving >50% conversion at ~180 °C and the lowest T_90_ among the tested series. Despite its relatively low specific surface area, Cu1-OMS-2 proved to be the most active catalyst, highlighting that surface area was not the determining factor for activity. Instead, O_2_-TPD analysis indicated that enhanced performance is associated with an increased contribution of surface and lattice oxygen species, promoted by the formation of Cu–O–Mn interactions within the cryptomelane lattice. The observed decline in activity at higher Cu loadings suggests the presence of an optimal dopant concentration and confirms that controlled Cu incorporation is a key strategy for enhancing the catalytic efficiency of OMS-2 materials.

## Figures and Tables

**Table 1:** Bulk (XRF) and surface (XPS) chemical composition and specific surface area of investigated catalysts.

Sample	S_BET_, m^2^ g^−1^	Normalized Values as Obtained from XRF *			Cu/Mn from XRF	Normalized Values as Obtained from XPS *				Cu/Mn from XPS
		**Mn**	**Cu**	**K**		**Mn**	**Cu**	**K**	O **	
OMS-2	66.9	8.00	-	0.88	-	8.00	-	0.88	17.12	-
Cu1-OMS-2	58.6	7.94	0.06	0.87	0.01	7.85	0.15	0.90	17.27	0.02
Cu2-OMS-2	59.6	7.90	0.10	0.82	0.01	7.85	0.15	0.88	17.25	0.02
Cu3-OMS-2	64.9	7.79	0.21	0.79	0.03	7.73	0.27	0.78	17.24	0.04

* molar % normalized to the sum of the manganese and dopant cations in the cryptomelane formula unit equal to 8. ** calculated from the total area of the O1s XPS peak.

**Table 2:** Comparison of the catalytic performance for toluene oxidation.

Catalyst	Process Parameters	T_50_, °C	T_90_, °C	Reference
CMO-1 nanostructured CuMn oxide	GHSV: 60,000 mL⸱g^−1^⸱h^−1^ Sample mass: 0.20 g Toluene concentration: 500 ppm	230	234	[40]
3Mn2Ce mixed oxide derived from MOF	GHSV: 30,000 mL⸱g^−1^⸱h^−1^ Sample mass: 0.10 g Toluene concentration: 1000 ppm	235	256	[42]
10Co/OMS-2 10 wt.% of cobalt over cryptomelane	GHSV: 60,000 mL⸱g^−1^⸱h^−1^ Sample mass: 0.05 g Toluene concentration: 2000 ppm	224	245	[29]
KMnO_4_-HT manganese oxide	GHSV: 60,000 mL⸱g^−1^⸱h^−1^ Sample mass: 0.10 g Toluene concentration: 500 ppm	226	237	[43]
CMO-400 CuMn mixed oxide calcined at 400 °C	GHSV: 30,000 mL⸱g^−1^⸱h^−1^ Sample mass: 0.20 g Toluene concentration: 1000 ppm	210	231	[44]
MnO_2_-CM	GHSV: 40,000 mL⸱g^−1^⸱h^−1^ Sample mass: 0.15 g Toluene concentration: 1000 ppm	197	207	[45]
Mn_1.82_Fe_0.18_O_3_	GHSV: 60,000 mL⸱g^−1^⸱h^−1^ Sample mass: 0.10 g Toluene concentration: 500 ppm	203	226	[46]
CuMnO_X_-HS	GHSV: 60,000 mL⸱g^−1^⸱h^−1^ Sample mass: 0.10 g Toluene concentration: 500 ppm	200	212	[47]
CuMnO_x_/0.03CeO_x_/CH-WH	GHSV: 40,000 mL⸱g^−1^⸱h^−1^ Sample mass: 0.15 g Toluene concentration: 500 ppm	200	210	[48]
Cu1-OMS-2	GHSV: 20,000 mL⸱g^−1^⸱h^−1^ Sample mass: 0.25 g Toluene concentration: 500 ppm	180	195	This study

## Data Availability

The original contributions presented in this study are included in the article/Supplementary Material. Further inquiries can be directed to the corresponding author.

## Supplementary Materials

The following supporting information can be downloaded at: https://www.mdpi.com/article/10.3390/ma19010159/s1, Figure S1: XPS spectra Mn 2p3/2 (1), K 2p (2), O 1s (3), Cu 2p3/2 (4) of investigated samples: OMS-2 (a), Cu1-OMS-2 (b), Cu2-OMS-2 (c), Cu3-OMS-2 (d). Table S1: Surface composition (atomic %) determined by fitting XPS spectra for OMS-2, Cu1-OMS-2, Cu2-OMS-2, and Cu3-OMS-2.; Table S2. Carbon balance during toluene combustion over investigated catalysts.
