# Supplementary material for: Enhanced Toluene Combustion over Cryptomelane Catalysts: Influence of Cu Doping on Physicochemical Properties and Catalytic Performance
Source: Materials (Basel). 2026 Jan 2;19(1):159. doi: 10.3390/ma19010159 (PMC12786640; doi:10.3390/ma19010159)
Supplement: Supplementary file 1 [file materials-19-00159-s001.zip › materials-4040650-supplementary.pdf]

Supplementary information

Enhanced toluene combustion over cryptomelane catalysts: Influence of Cu doping on physicochemical properties and catalytic performance

Jakub Mokrzycki[1*], Joanna Kryśniak-Czerwenka[2], Dorota Duraczyńska[2], Mateusz Marzec[3], Robert Karcz[2]

[1]AGH University of Krakow, Faculty of Energy and Fuels, al. A. Mickiewicza 30, 30-059 Krakow, Poland

[2]Jerzy Haber Institute of Catalysis and Surface Chemistry, Polish Academy of Sciences, Niezapominajek 8, 30-239 Krakow, Poland

[3]AGH University of Krakow, Academic Centre for Materials and Nanotechnology, al. A. Mickiewicza 30, 30-059 Krakow, Poland

*Corresponding author: Jakub Mokrzycki,

E-mail: jmokrzycki@agh.edu.pl

Tel.: +48-12-617-21-16

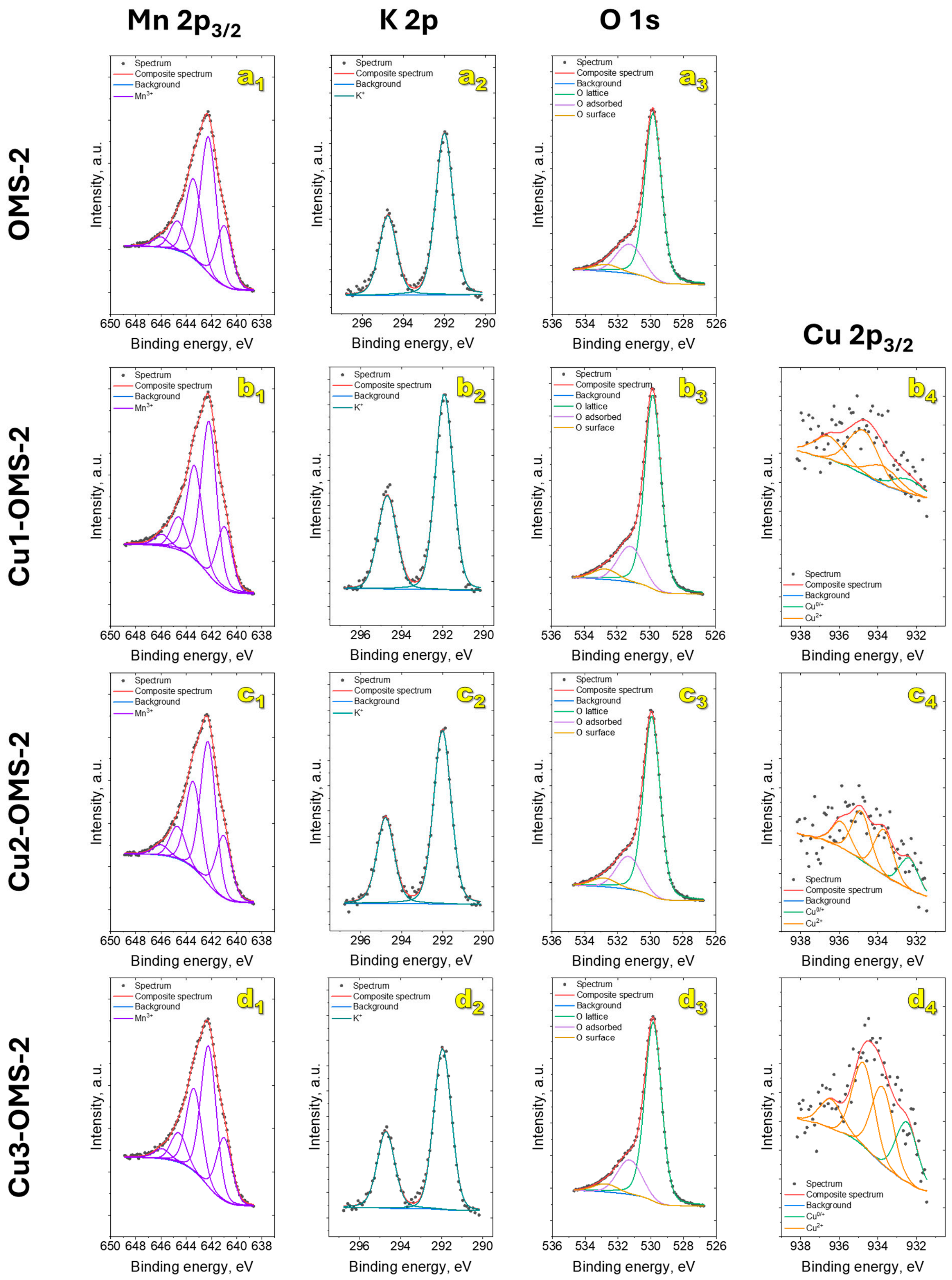

Figure S1. XPS spectra Mn $2p_{3/2}$ (1), K 2p (2), O 1s (3), Cu $2p_{3/2}$ (4) of investigated samples: OMS-2 (a), Cu1-OMS-2 (b), Cu2-OMS-2 (c), Cu3-OMS-2 (d).

Table S1. Surface composition (atomic %) determined by fitting XPS spectra for OMS-2, Cu1-OMS-2, Cu2-OMS-2, and Cu3-OMS-2.

Element	O			K	Mn	Cu
	$O_{lattice}$	$O_{adsorbed}$	$O_{surface}$			
Binding energy, eV	529.9	531.3	535.0	292.0	641.0	933.3
OMS-2	43.5	10.7	2.5	2.9	26.5	0
Cu1-OMS-2	42.3	10.5	3.1	2.9	25.4	0.5
Cu2-OMS-2	43.4	10.5	2.8	2.9	25.8	0.5
Cu3-OMS-3	43.6	10.9	2.6	2.6	25.6	0.9

Carbon balance during catalytic test

The carbon balance was calculated using following reaction equation:
$C_7H_8+9O_2=7CO_2+4H_2O$
Concentration of carbon at inlet: $C_{C,in} = 7*n_{C_7H_8,in}$
Concentration of carbon at outlet: $C_{C,out} = n_{CO_2}+7n_{C_7H_8,out}$
Our carbon balance is ratio of $C_{C,in}/C_{C,out}$.
A value close to 1 shows that toluene is converted mainly to carbon dioxide. Values lower than 1 were recorded at low temperatures when toluene was being adsorbed at catalyst surface. Prolonged stabilization of catalyst allows to saturate the surface with toluene and reach value of 1. The apparent increase in ratio at higher temperatures >180 °C may result from burning of adsorbed toluene as well as changes in gas stream density induced by increasing temperature of reactor.

Table S2. Carbon balance during toluene combustion over investigated catalysts.

Catalyst	Temperature, °C	$C_{C,in}/C_{C,out}$	Remarks
OMS-2	150	1.01	Conversion to CO_2
	180	0.99	Conversion to CO_2
	190	1.01	Conversion to CO_2
Cu1-OMS-2	150	0.54	Sorption of toluene, no CO_2 formed
	170	1.00	Conversion to CO_2
	180	1.19	Burning of adsorbed toluene
	190	1.15	Conversion to CO_2
	200	1.10	Conversion to CO_2
Cu2-OMS-2	150	0.79	Sorption of toluene
	170	1.00	Conversion to CO_2
	180	1.15	Burning of adsorbed toluene
	190	1.10	Conversion to CO_2
	200	1.08	Conversion to CO_2
Cu3-OMS-2	150	0.49	Sorption of toluene
	170	0.94	Conversion to CO_2
	180	1.09	Burning of adsorbed toluene
	190	1.10	Conversion to CO_2
	200	1.16	Conversion to CO_2
